# Why Iron Deficiency in Acute Heart Failure Should Be Treated: A Real-World Clinical Practice Study

**DOI:** 10.3390/life12111828

**Published:** 2022-11-09

**Authors:** Raquel López-Vilella, Víctor Donoso Trenado, Pablo Jover Pastor, Ignacio Sánchez-Lázaro, Luis Martínez Dolz, Luis Almenar Bonet

**Affiliations:** 1 Heart Failure and Transplantation Unit, Hospital Universitario y Politécnico La Fe, 46026 Valencia, Spain; 2 Department of Cardiology, Hospital Universitario y Politécnico La Fe, 46026 Valencia, Spain; 3 Centro de Investigación Biomédica en Red de Enfermedades Cardiovasculares (CIBERCV), Instituto de Salud Carlos III, 28029 Madrid, Spain

**Keywords:** acute heart failure, iron deficiency, ferric carboxymaltose, preserved ejection fraction, reduced ejection fraction, morbidity, mortality

## Abstract

**Background.** This study aims to determine whether the administration of ferric carboxymaltose (FCM) in patients with acute heart failure (AHF) and iron deficiency (ID) improves morbidity and mortality. **Methods.** We studied 890 consecutive patients admitted for AHF. Patients were divided into six groups according to reduced left ventricular ejection fraction (HFrEF) or preserved (HFpEF), presence of ID, and administration of FCM. Emergency visits, re-admissions, and all-cause mortality were assessed at 6 months. **Results.** The overall prevalence of ID was 91.2%. In the HFrEF group, no differences were found in isolated events when patients with untreated vs. treated ID were compared, while differences were found in the combined event rate (*p* = 0.049). The risk calculation showed an absolute risk reduction (ARR) of 10% and relative risk reduction (RRR) of 18%. In HFpEF there was a positive trend with regard to the combined event (*p* = 0.107), with an ARR of 9% and an RRR of 15%. The number of patients we needed to treat to prevent a combined event was 10.5 in HFrEF and 10.8 in HFpEF. **Conclusions.** FCM in AHF reduced the combined event rate of emergency visits, re-admission, and all-cause death at 6 months in HF with left ventricular ejection fraction <50%, and showed a positive trend in HFpEF.

## 1. Introduction

Heart failure (HF) is a disease that significantly affects quality of life (QoL) and reduces survival [1,2,3]. The incidence of HF continues to rise due to the increasing longevity of the population and the comorbidities that usually accompany aging [2]. Consequently, healthcare systems are required to invest increasing resources for better management and control of this disease [1,2], while the treatment of comorbidities associated with HF is crucial to improving the prognosis and QoL of these patients [4,5]. HF is a clinical syndrome that interferes with iron metabolism and may cause a deficiency of this trace element. The treatment of iron deficiency (ID) in HF has been one of the most widely studied strategies in the recent past [6,7,8], and the administration of intravenous (i.v.) iron in ambulatory HF patients with reduced ejection fraction (HFrEF) has been found to improve HF symptoms, QoL, and left ventricular ejection fraction (LVEF), with no significant side effects [6,7,8,9]. Evidence has recently shown that i.v. administration of ferric carboxymaltose (FCM) before hospital discharge improves symptoms and reduces re-admissions in patients with ID admitted for acute HF (AHF) [10], but there is little scientific evidence in real-world clinical practice. The potential benefit of FCM should be evaluated in a larger population group with AHF that includes patients with HFrEF and HFpEF, as occurs in the real-world setting.

The hypothesis of this study was that i.v. administration of FCM to patients with AHF and ID could reduce morbidity and mortality in both HFrEF and HFpEF.

The primary objective of this routine clinical practice study was to analyze the effect of FCM administration on the likelihood of re-admission due to cardiac decompensation, emergency visits due to clinical instability, and short-term all-cause mortality (6 months) in patients admitted for HF, regardless of their ejection fraction.

## 2. Materials and Methods

### 2.1. Study and Patient Cohort

**Figure 1:**
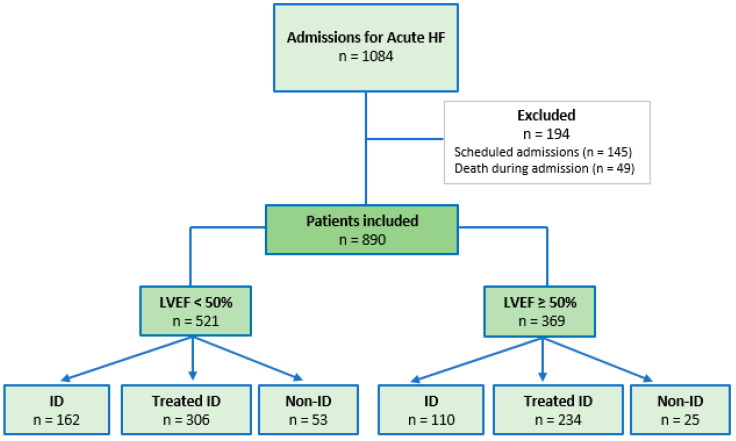
**Flow chart of study patient recruitment.** The observational analysis includes a total of 6 study groups. HF, heart failure; ID, iron deficiency; LVEF, left ventricular ejection fraction.

We recruited 1084 patients admitted consecutively with a diagnosis of decompensated AHF in any of its forms (acute pulmonary edema, systemic congestion, mixed congestion [pulmonary and systemic], and low cardiac output) to the cardiology department of a tertiary referral hospital. Patients transferred from other hospital departments and those who died during admission were excluded. A total of 890 patients were included in the retrospective analysis over a 3-year period (May 2018–May 2021). Follow-up was 6 months (Figure 1).

Patients were divided into 6 groups depending on 3 variables: LVEF (preserved [≥50%] vs. reduced [<50%]); presence of ID (ID vs. non-ID); and administration of FCM during hospitalization (treated vs. untreated). Treatment with FCM during admission was decided by the attending physician. Thus, the groups analyzed were: (1) HFrEF with ID; (2) HFrEF with ID treated with FCM; (3) HFrEF without ID; (4) HFpEF with ID; (5) HFpEF with ID treated with FCM; and (6) HFpEF without ID. We used the cut-off point of 50% left ventricular ejection fraction following the most recent clinical practice guidelines and also following the outline of the main clinical trial of iron deficiency in acute heart failure [3,10].

Clinical, laboratory, echocardiographic, and treatment variables were analyzed in each study group. The number of hospital emergency visits, number of re-admissions, and mortality were recorded after hospital discharge and during the follow-up period. Risk differences between treated and untreated patients were also analyzed. All patients admitted for decompensated HF are usually followed up for 6 months in our hospital, so this period was chosen as the study endpoint.

Quantitative assessment of LVEF and qualitative analysis of right ventricular function were performed by echocardiogram during admission. HFpEF was defined according to European Society of Cardiology (ESC) guidelines for the diagnosis and treatment of acute and chronic HF [3]. Patients were diagnosed with HFpEF if they had signs and symptoms of HF with LVEF ≥ 50%, raised natriuretic peptide levels (NT-proBNP), and at least one additional criterion (relevant structural heart disease or diastolic dysfunction).

All patients had a predefined laboratory test panel performed on the day following admission that included iron parameters (ferritin levels and transferrin saturation index [TSAT]) [11,12]. ID was diagnosed and treated with FCM according to the criteria established in the ESC HF guidelines (3) (ferritin levels < 100 µg/L or 100–300 µg/L with TSAT < 20%). The FCM dose administered was 1000 mg diluted in 250 cc of 9% saline infused over 30 min or the same dose diluted in 100 cc infused over 15 min. For patients weighing < 50 kg, 500 mg was administered in the same diluent and over the same time. For patients with hemoglobin levels ≥ 14 g/dL, the FCM dose administered was 500 mg. This study was carried out in accordance with the Declaration of Helsinki and approved by the Ethics Committee of the Hospital Universitario y Politécnico La Fe, Valencia (Spain).

### 2.2. Statistical Analysis

Qualitative variables were expressed as percentages and quantitative variables as means and standard deviation (SD) or as medians and interquartile ranges (IQRs; 25–75%) in the case of *p* < 0.05 after confirming normality with the Kolmogorov–Smirnov (Z) test. The association between quantitative variables with normal distribution was analyzed using the Student’s *t*-test, while the χ^2^ test or Wilcoxon rank test for two related samples was used for the remaining variables.

A *p*-value of < 0.05 was taken as significant. The absolute risk reduction (ARR), relative risk reduction (RRR), and number of patients needed to treat (NNT) were calculated using preconfigured formulas. Statistical analysis was performed using SPSS Statistics software Version 27^®^ and Stata Statistics/Data analysis 16.1, serial number 501606323439.

## 3. Results

### 3.1. Prevalence and Clinical Profile of Patients

**Figure 2:**
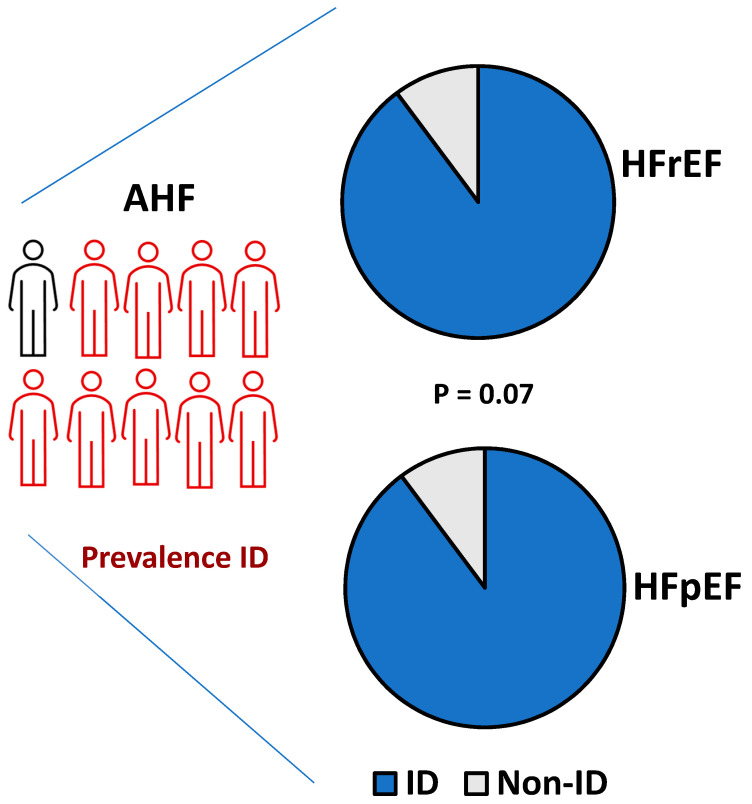
High prevalence of iron deficiency in the study population. AHF, acute heart failure; HFpEF, heart failure with preserved ejection fraction; HFrEF, heart failure with reduced ejection fraction; ID, iron deficiency.

ID was very common in the series of patients admitted for AHF who were included in the study (91.2%). In the HFrEF and HFpEF subgroups, prevalence was 89.8% and 93%, respectively. These values were close to statistical significance (*p* = 0.07) (Figure 2). The prevalence of anemia (Hemoglobine (Hb) < 12 was 432 patients (48.5%). In HFrEF it was 239/521 (46%), and in HFpEF it was 193/369 (52%).

In the HFrEF group, patients without ID were on average 5 years younger than those with ID, which may explain the lower incidence of comorbidities such as diabetes mellitus (DM) and hypertension (HT). A slightly higher LVEF was also detected in this patient group (Table 1). Following the same trend, the analysis of patients with HFpEF revealed some differences between the clinical characteristics of the subgroups. Thus, patients without ID were on average 6 years younger and also had a lower prevalence of DM (Table 2). No differences were noted between clinical laboratory parameters analyzed 24 h post-admission, except for the iron levels, which were normal in the non-ID group (Table 3 and Table 4). An isolated significant difference in the treatment received at discharge by the HFrEF group (between subgroups with and without ID) was detected but was not considered clinically relevant (angiotensin-converting enzyme inhibitors [ACEIs] and angiotensin II receptor blockers [ARBs]) (Table 3).

### 3.2. Effect of FCM Treatment on Morbidity and Mortality

**Figure 3:**
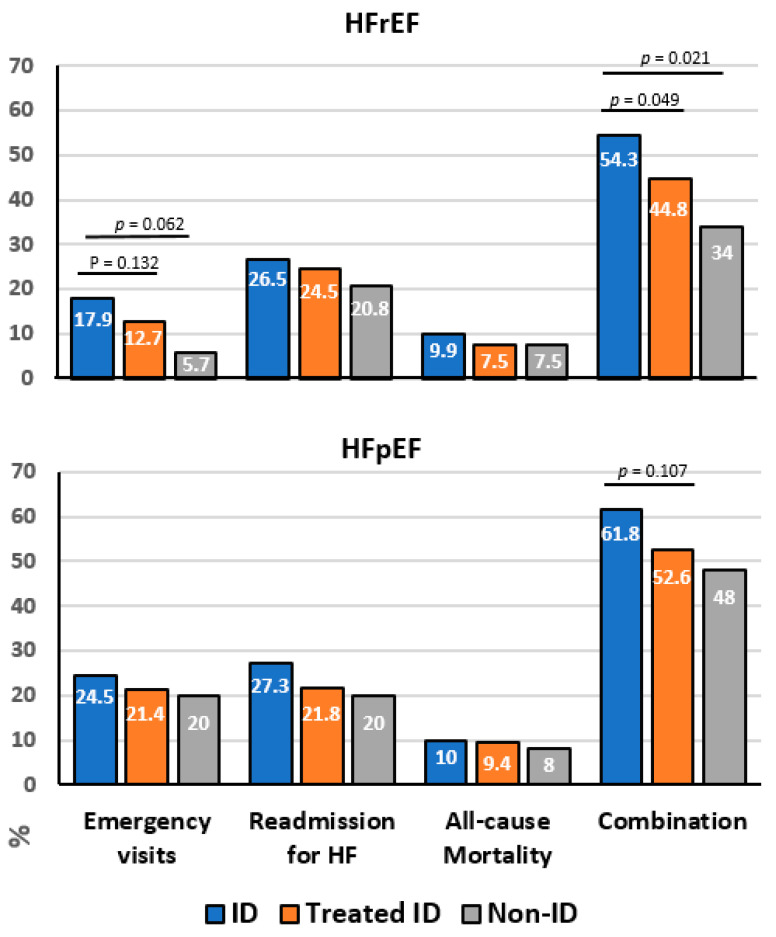
**FCM treatment reduces morbidity and mortality events.** Data are expressed as % cases per group. The 3 ID vs. treated ID groups were analyzed. HF, heart failure; HFpEF, heart failure with preserved ejection fraction; HFrEF, heart failure with reduced ejection fraction; ID, iron deficiency.

A comparative analysis between the 3 patient groups with HFrEF showed no significant differences when the study objectives were examined separately (emergency visits, re-admission for HF, and all-cause mortality). However, statistically significant differences were found in the comparison of the combined event between the FCM-treated ID vs. untreated ID groups (*p* = 0.049). Nevertheless, the improvement in the treated group did not reach the values of the parameters analyzed in the non-ID group. Only the mortality rate was almost similar among the 3 patient groups (Figure 3, Table 5).

In the HFpEF group, no significant differences were found in the variables taken individually or in the combined event rate, although there was a trend toward a reduction in the combined event rate in the ID group treated with FCM vs. the untreated group (*p* = 0.107). The values for the combined event presented by non-ID patients were not achieved in this type of HF either (Figure 3, Table 5).

**Figure 4:**
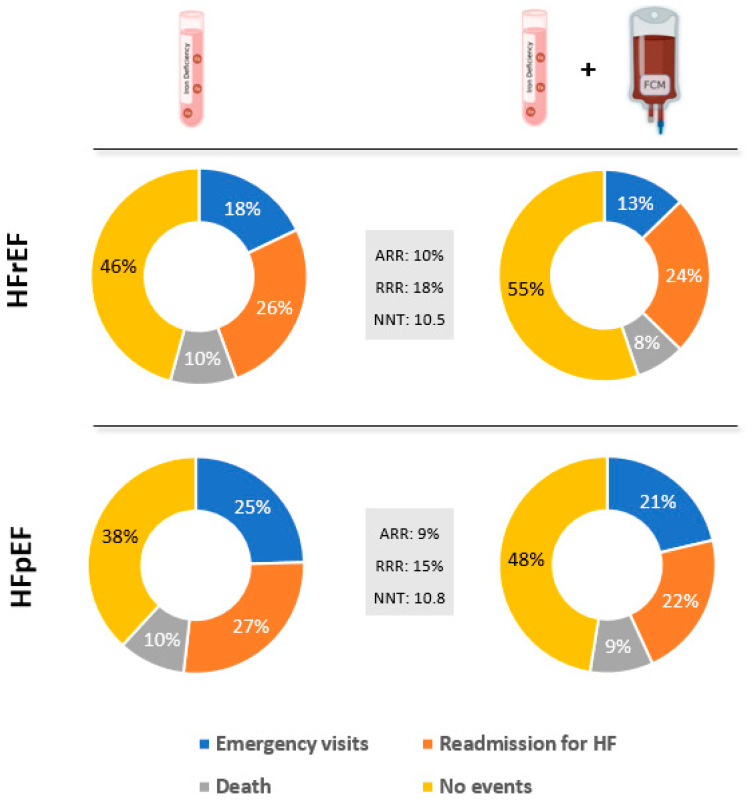
**Impact of ferric carboxymaltose treatment on risk reduction and the number of patients needed to treat.** Percentage of iron-deficient patients with combined events by ejection fraction and treatment administration. ARR, absolute risk factor; FCM, ferric carboxymaltose; HF, heart failure; HFpEF, heart failure with preserved ejection fraction; HFrEF, heart failure with reduced ejection fraction; ID, iron deficiency; NNT, number needed to treat, RRR, relative risk factor.

To analyze the clinical relevance of FCM treatment in patients with ID hospitalized for AHF, the ARR and RRR were calculated for each of the study objectives, alone and also in combination. Thus, in HFrEF, the greatest ARR and RRR were recorded for the number of emergency visits, while in HFpEF, the greatest risk reduction was obtained for re-admissions for HF. The NNT with FCM to prevent a combined event was 10.5 in HFrEF and 10.8 in HFpEF (Figure 4, Table 6), suggesting that treatment with FCM in the scenario analyzed had a high clinical impact.

## 4. Discussion

The prevalence of ID in patients with chronic HF is very high [13,14,15,16,17]. The ESC clinical guidelines for HF recently incorporated the recommendation to administer FCM in order to replete iron stores and enhance its use by the body in an acute setting [3]. In this context, our study shows that treatment with FCM in ID patients with AHF reduces the percentage of events at 6 months (emergency visits, re-admission for HF, and death), particularly in patients with HFrEF. There are virtually no large studies at present that have examined the effectiveness of administering FCM in an acute decompensation setting, either in HFrEF or HFpEF.

Following the results of the AFFIRM-AHF study, the administration of FCM before hospital discharge was recommended to improve symptoms and reduce re-admissions [10]. In our study, the administration of FCM in ID patients was at the discretion of the attending physician, since at the time of recruitment, no evidence was available on the benefits of iron administration during admission or in patients with preserved LVEF. The use of FCM meets treatment standards, as it is the most extensively studied i.v. preparation and there are currently no recommendations for the use of oral iron [18].

Our analyses found a high prevalence of ID in patients admitted for AHF, which was higher in HFpEF than in HFrEF (93.2% vs. 89.8%) and revealed a higher prevalence than has been reported thus far (around 50%) [13,14,16,17,19]. Other studies have established that the prevalence of ID in the acute setting is higher than outside decompensation periods, with values more similar to those obtained in our study (72–83%) [19]. In line with our findings, some authors point to a higher prevalence of ID in HFpEF of about 73% in patients in stable conditions [20]. Others, however, estimate a similar prevalence between non-decompensated HFrEF and HFpEF [21,22]. Our study provides new evidence on the incidence of ID in patients with acute decompensated HFrEF, which has been poorly studied to date. Moreover, a higher incidence of ID has been observed in anemic patients (even though it is an independent condition of anemia), and also in women, diabetics, more advanced functional class, greater burden of comorbidities, and higher levels of C-reactive protein and NT-proBNP. These conditions are very common in HFpEF [13,17,22]. Multiple overlapping mechanisms are theorized to lead to ID in HF: Inflammation from chronic HF, low flow states and associated early satiety lead to malnutrition and thus poor iron intake, edema in gastrointestinal walls, and chronic inflammation [23].

A subgroup analysis of non-ID patients and patients with ID treated or untreated within the HFrEF and HFpEF populations allowed us to study the characteristics and behavior of these subgroups. The mean age of the HFrEF patient group was close to 70 years, similar to cohorts included in other studies of similar characteristics [10,21]. In our series, non-ID patients were significantly younger, which contrasts with the results published in other papers, in which no age differences were found between patients according to their iron levels [21,22]. In the HFrEF group, we found a higher prevalence of men and a higher frequency of ischemic heart disease, as described in other studies [24]. With regard to cardiovascular risk factors (CVRF), ID patients had a higher prevalence of HT and DM, which in the case of DM is consistent with the literature [22,25]. As in previous studies, no significant differences were observed in other comorbidities [21,22]. Our non-ID patients had a slightly higher LVEF than patients with ID, in line with the results of the subanalysis of the Myocardial-IRON study, which showed a better iron status with better ventricular function in patients with HFrEF [26,27].

In terms of laboratory variables, no significant differences were observed between subgroups of patients with HFrEF, with renal function, hemoglobin and transaminase values similar to those recorded in other studies [6,7,8,10]. It should be noted that NT-proBNP levels in our series were similar to those observed in the AFFIRM-AHF study [10] but higher than those reported in other literature [6,26,28,29], probably due to the acute decompensation situation in the study population. Our analysis confirmed lower than expected rates in the use of beta-blockers and mineralocorticoid receptor agonists (MRA) as baseline treatment, which may be due to the high number of patients diagnosed with de novo HF (> 30%). In general, the use of these drugs is similar to that of other cohorts of patients with acute HF [10,23], and different from the medication regimen followed by patients with chronic HF [6,7,8,28].

The HFpEF population in our study was generally older on average than the HFrEF population and the prevalence of women was higher, which is common in populations with this disease. Similarly, the non-ID population were younger on average, confirming the findings of other studies [22]. Patients with HFpEF have a lower frequency of ischemic heart disease, with valvular and hypertensive heart disease being more common, and there are no differences between subgroups [20,30]. In terms of CVRF, only a trend toward a higher prevalence of DM was detected in ID patients, similar to published data [22]. No differences were found in LVEF (around 60%) or right ventricular function, as previously described in patients with HFpEF [20,21,22]. No significant differences were observed either in the laboratory variables between groups. However, higher NT-proBNP levels than those generally recorded in the few studies that included patients with acute HFpEF were confirmed [31,32]. With regard to baseline treatment, no differences were found between subgroups, nor were notable differences found with respect to treatment reported in the literature for this very heterogeneous disease, in which the use of diuretics predominates [20,21,22].

Many studies have been conducted in patients with chronic or stable HFrEF, in which treatment with FCM has shown improvement in functional capacity and exercise capacity [6,7,27], and even effects on ventricular remodeling [29,33,34] and a reduction in hospital admissions for HF, with no clear impact on mortality [7,8,15,16,20,35]. The evidence available in acute HFrEF is relatively recent. The AFFIRM-AHF study and a subanalysis of its results showed that treatment with FCM was safe, reduced the risk of HF hospitalizations, had no effect on cardiovascular mortality, and improved QoL [10,36]. The PRACTICE-ASIA-AHF study reported that FMC improved functional capacity [24]. In our patient series, administration of FCM in the acute phase had a greater effect on patients with HFrEF. In particular, a reduction in combined events (emergency visits, HF re-admissions, and mortality) between treated and untreated ID patients was confirmed, similar to the findings of the AFFIRM-AHF study, which found no differences in mortality but did find differences in the combined event rate and in HF admissions at the 1-year follow-up [10]. In our analysis, we also observed a trend toward a reduction in admissions and emergency visits in non-ID patients and in treated ID patients that would probably have become significant with a larger sample size or longer follow-up time.

There is little evidence on ID in HFpEF, especially in the acute phase. Chronic-phase studies associate the ID in HFpEF with worse functional class, exercise capacity and QoL, while not demonstrating any effect on hospitalization or mortality rates [13,14,20,22,37,38]. However, the presence of ID in patients admitted for HF was related to the rate of re-admissions, independently of the LVEF [39]. Even so, the progression of ID is known to carry a higher risk of HF admission and all-cause mortality [40]. A study carried out in acute-phase patients, in which subjects with HFpEF accounted for 55% of the sample, found that ID was associated with a longer hospital stay regardless of other factors such as comorbidities or proinflammatory status, which was not observed in patients with HFrEF [21]. To date, there are no robust studies on treatment with FCM in patients with HFpEF, although some evidence suggests that it could improve functional status and LVEF [9]. However, more conclusive results are expected for this patient group with the completion of the FAIR-HFpEF trial (NCT03074591). In our study, similarly to that described in the evidence, no significant differences were observed in the combined event rate or in the individual events when we compared patients with and without ID, treated with FCM and untreated. Nevertheless, we noted a trend toward a reduction in events that should be validated in longer-term studies.

Risk-reduction analysis for the combined event in both the HFrEF and HFpEF groups determined an NNT of 10, suggesting that treatment with FCM provides a significant potential benefit in the setting of decompensated HF.

The main limitation of our study is its retrospective nature. Nevertheless, data from routine clinical practice provide very relevant information, as they reflect the reality of patient management. Furthermore, the number of patients included is substantial and the subgroups are generally well balanced. The study was carried out in a single hospital center, which may imply a lack of diversity, but on the other hand, the data entry was concurrent with patient admission and was always performed by the same experts, so errors are minimized. The study was not randomized, and the administration of FCM was at the discretion of each patient’s attending physician. A 6-month follow-up period was chosen as the standard time criterion for monitoring patients admitted for decompensated HF, but this may be insufficient to assess effects on mortality.

Despite these limitations, this study is the first to analyze the effectiveness of FCM in subjects with decompensated AHF with both reduced and preserved ejection fractions in the real-world setting, and with a significant number of patients. In addition, another strength of the study is that a subgroup comparison was made to verify whether treatment with FCM in patients with ID could normalize the risk of morbidity and mortality to values found in non-ID patients.

## 5. Conclusions

Administration of FCM in patients with decompensated AHF and ID is useful and effective in reducing the combined event (emergency visits, re-admission for HF, and all-cause death) at 6 months. The NNT to prevent an event is 10, in both HFrEF and HFpEF. This real-world evidence should be implemented as soon as possible in all patients admitted for decompensated HF, regardless of LVEF.

## Figures and Tables

**Table 1:** **Baseline characteristics of HFrEF study patients.** Data are expressed as % of cases and mean ± SD for normally distributed variables (*). *p* < 0.05 was considered statistically significant.

	ID *n* = 162	Treated ID *n* = 306	No ID *n* = 53	*p*
**Patient history (*n*, %)**				
Age (years) (*)	73.0 ± 12.1	73.4 ± 10.4	68.2 ± 12.3	***0.007***
Male	109 (67.3)	211 (69.0)	34 (64.2)	*0.769*
*Baseline heart disease (n, %)*				
IHD	63 (38.9)	122 (40.0)	21 (39.6)	*0.979*
VHD	26 (16.0)	49 (16.0)	8 (15.1)	*0.982*
AF	15 (9.3)	15 (4.9)	4 (7.5)	*0.183*
DCM	35 (21.6)	70 (22.9)	12 (22.6)	*0.952*
HT	17 (10.5)	40 (13.1)	7 (13.2)	*0.705*
Other	6 (3.7)	10 (3.3)	2 (3.8)	*0.812*
*History (n, %)*				
CVS	36 (22.2)	61 (19.9)	8 (15.1)	*0.527*
HT	125 (77.2)	229 (74.8)	29 (54.7)	***0.004***
Dyslipidemia	100 (61.7)	180 (58.8)	24 (45.3)	*0.105*
DM	96 (59.3)	170 (55.6)	21 (39.6)	***0.043***
Smoking	23 (14.2)	35 (11.4)	4 (7.5)	*0.399*
Alcoholism	6 (3.7)	19 (6.2)	2 (3.8)	*0.451*
COPD	26 (16.0)	58 (19.0)	8 (15.1)	*0.644*
SAHS	24 (14.8)	43 (14.1)	9 (17.0)	*0.852*
Obesity (BMI > 30 kg/m^2^)	39 (24.1)	71 (23.2)	12 (22.6)	*0.938*
Renal failure	37 (22.8)	60 (19.6)	10 (18.9)	*0.678*
Hypothyroidism	7 (4.3)	28 (9.2)	3 (5.7)	*0.143*
AF	82 (50.6)	156 (51.0)	29 (54.7)	*0.865*
Stroke	15 (9.3)	35 (11.4)	4 (7.5)	*0.593*
PVD	16 (9.9)	36 (11.8)	3 (5.7)	*0.387*
**Clinical characteristics (*n*, %)**				
No. of previous admissions (*)	0.8 ± 0.6	0. 8 ± 0.6	0.7 ± 0.5	*0.506*
de novo HF	63 (38.9)	103 (33.7)	18 (34.01)	*0.518*
*FC (NYHA)*				
I	37 (22.8)	65 (21.3)	11 (20.8)	*0.910*
II	79 (48.8)	142 (46.4)	25 (47.2)	*0.959*
III	45 (27.8)	94 (30.7)	17 (32.1)	*0.754*
IV	1 (0.6)	5 (1.6)	0 (0.0)	*0.438*
*Cause of decompensation*				
Arrhythmia	35 (21.6)	64 (20.9)	12 (22.6)	*0.955*
Infectious	18 (11.1)	36 (11.8)	6 (11.4)	*0.977*
Ischemic	15 (9.3)	26 (8.5)	5 (9.4)	*0.551*
Disease progression	59 (36.4)	113 (36.9)	20 (37.7)	*0.984*
Unknown	30 (18.5)	67 (21.9)	8 (15.1)	*0.430*
HT	5 (3.1)	8 (2.6)	2 (3.8)	*0.881*
*Hemodynamic pattern*				
Pulmonary congestion	113 (69.8)	203 (66.3)	35 (66.0)	*0.737*
Systemic pulmonary congestion	17 (10.5)	30 (9.8)	7 (13.2)	*0.753*
Systemic congestion	30 (18.5)	69 (22.5)	9 (17.0)	*0.460*
Low output	2 (1.2)	4 (1.3)	2 (3.8)	*0.376*
**Echocardiography (*n*, %)**				
LVEF (*)	32.7 ± 9.6	33.4 ± 10.2	37.3 ± 10.4	***0.014***
*RV function*				
Normal	112 (69.1)	179 (58.5)	36 (68.0)	*0.055*
Mild depression	11 (6.8)	24 (7.8)	4 (7.5)	*0.919*
Moderate depression	24 (14.8)	43 (14.1)	8 (15.1)	*0.964*
Severe depression	15 (9.3)	18 (5.9)	5 (9.4)	*0.335*

AF, atrial fibrillation; BMI, body mass index; COPD, chronic obstructive pulmonary disease; CVS, cardiovascular surgery; DCM, dilated cardiomyopathy; DM, diabetes mellitus; FC, functional class; HF, heart failure; HT, hypertension; IHD, ischemic heart disease; LVEF, left ventricular ejection fraction; NYHA, New York Heart Association functional classification of the HF; PVD, peripheral vascular disease; RV, right ventricle; SAHS, sleep apnea-hypopnea syndrome; SD, standard deviation; VHD, valvular heart disease.

**Table 2:** **Baseline characteristics of HFpEF study patients.** Data are expressed as % of cases and mean ± SD for normally distributed variables (*). *p* < 0.05 was considered statistically significant.

	ID *n* = 110	Treated ID *n* = 234	No ID *n* = 25	*p*
**Patient history (*n*, %)**				
Age (years) (*)	77.9 ± 9.9	79.9 ± 7.3	72.9 (9.3)	***<0.0001***
Male	33 (30.0)	83 (35.5)	7 (28.0)	*0.509*
*Baseline heart disease (n, %)*				
IHD	4 (3.6)	18 (7.7)	2 (8.0)	*0.46*
VHD	53 (48.2)	102 (43.6)	10 (40.0)	*0.644*
AF	12 (10.9)	20 (8.5)	3 (12.0)	*0.714*
DCM	0 (0.0)	0 (0.0)	0 (0.0)	*1*
HT	33 (30.0)	85 (36.3)	9 (40.0)	*0.508*
Other	8 (7.3)	9 (3.8)	1 (4.0)	*0.379*
*History (n, %)*				
CVS	14 (12.7)	42 (17.9)	4 (16.0)	*0.472*
HT	95 (86.4)	213 (91.1)	20 (80.0)	*0.150*
Dyslipidemia	59 (53.6)	135 (57.7)	15 (60.0)	*0.732*
DM	46 (41.8)	73 (31.2)	5 (20.0)	***0.05***
Smoking	12 (10.9)	12 (5.1)	2 (8.0)	*0.146*
Alcoholism	7 (6.4)	13 (5.6)	1 (4.0)	*0.871*
COPD	24 (21.8)	54 (23.1)	5 (20.0)	*0.921*
SAHS	15 (13.6)	44 (18.8)	4 (16.0)	*0.489*
Obesity (BMI > 30 kg/m^2^)	28 (25.5)	45 (19.2)	5 (20.0)	*0.415*
Renal failure	42 (38.2)	75 (32.1)	10 (40.0)	*0.383*
Hypothyroidism	16 (14.5)	36 (15.4)	4 (16.0)	*0.973*
AF	74 (67.3)	163 (69.7)	20 (80.0)	*0.458*
Stroke	16 (14.5)	36 (15.4)	4 (16.0)	*0.973*
PVD	1 (0.9)	3 (1.3)	0 (0.0)	*0.822*
**Clinical characteristics (*n*, %)**				
No. of previous admissions (*)	0.9 ± 0.5	0.9 ± 0.7	0.8 ± 0.6	*0.753*
de novo HF	42 (38.2)	75 (32.1)	10 (40.0)	*0.446*
*FC (NYHA)*				
I	11 (10.0)	18 (7.7)	3 (12.0)	*0.645*
II	68 (61.8)	142 (60.7)	15 (60.0)	*0.975*
III	22 (20.0)	53 (22.6)	5 (20.0)	*0.828*
IV	9 (8.2)	21 (9.0)	2 (8.0)	*0.963*
*Cause of decompensation*				
Arrhythmia	21 (19.1)	51 (21.8)	4 (16.0)	*0.711*
Infectious	3 (2.7)	9 (3.8)	2 (8.0)	*0.459*
Ischemic	2 (1.8)	4 (1.7)	2 (8.0)	*0.116*
Disease progression	52 (47.3)	115 (49.1)	12 (48.0)	*0.984*
Unknown	6 (5.5)	8 (3.4)	1 (4.0)	*0.672*
HT	26 (23.6)	41 (17.5)	4 (16.0)	*0.371*
*Hemodynamic pattern*				
Pulmonary congestion	76 (69.1)	160 (68.4)	18 (72.0)	*0.931*
Systemic pulmonary congestion	21 (19.1)	48 (20.5)	5 (20.0)	*0.954*
Systemic congestion	13 (11.8)	22 (9.4)	2 (8.0)	*0.738*
Low output	0 (0.0)	4 (1.7)	0 (0.0)	*0.832*
**Echocardiography (*n*, %)**				
LVEF (*)	63.2 ± 7.5	62.0 ± 7.0	60.0 ± 6.3	*0.094*
*RV function*				
Normal	82 (74.5)	174 (74.4)	20 (80.0)	*0.824*
Mild depression	19 (17.3)	44 (18.8)	5 (20.0)	*0.414*
Moderate depression	8 (7.3)	10 (4.3)	0 (0.0)	*0.234*
Severe depression	1 (0.9)	6 (2.6)	0 (0.0)	*0.445*

AF, atrial fibrillation; BMI, body mass index; COPD, chronic obstructive pulmonary disease; CVS, cardiovascular surgery; DCM, dilated cardiomyopathy; DM, diabetes mellitus; FC, functional class; HF, heart failure; HT, hypertension; IHD, ischemic heart disease; LVEF, left ventricular ejection fraction; NYHA, New York Heart Association functional classification of the HF; PVD, peripheral vascular disease; RV, right ventricle; SAHS, sleep apnea-hypopnea syndrome; SD, standard deviation; VHD, valvular heart disease.

**Table 3:** **Analytical and pharmacological HFrEF profile.** Data are expressed as % of cases and median ± interquartile range for non-normally distributed variables (#). *p* < 0.05 was considered statistically significant.

	ID *n* = 162	Treated ID *n* = 306	No ID *n* = 53	*p*
**Laboratory tests on admission (#)**				
Urea (mg/dL)	40.0 (46.0)	46.0 (85.0)	39.0 (42.0)	*0.614*
Creatinine (mg/dL)	1.03 (0.44)	1.06 (1.36)	1.08 (1.74)	*0.953*
GFR (mL/min/1.73 m^2^)	58.6 (31.0)	56.0 (69.9)	58.0 (50.4)	*0.893*
Bilirubin (mg/dL)	1.0 (0.5)	1.1 (0.6)	0.9 (0.5)	*0.863*
AST (U/L)	23.0 (9.0)	21.0 (14.0)	22.8 (10.1)	*0.203*
ALT (U/L)	22.6 (18.8)	23.0 (17.3)	24.0 (19.1)	*0.885*
TnT(u) (ng/mL)	45.9 (28.3)	44.0 (36.7)	48.1 (29.6)	*0.658*
NT-proBNP (pg/mL)	5762 (3870)	5398 (3207)	6296 (2748)	*0.158*
Sodium (mEq/L)	140.5 (7.0)	142.0 (7.3)	151.5 (7.1)	*0.101*
Potassium (mEq/L)	4.2 (0.8)	4.1 (1.4)	3.9 (0.7)	*0.270*
Hemoglobin (g/dL)	13.9 (2.0)	13.2 (4.9)	13.4 (4.1)	*0.219*
Hematocrit (%)	40.6 (7.8)	40.7 (11.9)	40.2 (10.4)	*0.951*
Uric acid (mg/dL)	8.8 (4.7)	8.5 (4.4)	8.6 (3.7)	*0.785*
Cholesterol-HDL (mg/dL)	42.1 (23.3)	43.0 (16.3)	44.5 (17.9)	*0.713*
Cholesterol-LDL (mg/dL)	79.2 (24.7)	74.0 (42.6)	76.5 (21.6)	*0.332*
Triglycerides (mg/dL)	133.5 (84.1)	148.7 (62.0)	151.5 (79.4)	*0.068*
Ferritin (ng/mL)	166.1 (135.2)	156.0 (89.1)	531.5 (223.2)	***<0.0001***
TSAT (%)	16.3 (6.0)	18.0 (10.9)	25.0 (16.1)	***<0.0001***
HbA1c (%)	6.3 (0.8)	6.3 (0.9)	6.5 (0.6)	*0.263*
CA125 (U/mL)	73.0 (62.9)	73.9 (60.6)	72.4 (69.2)	*0.980*
**Discharge treatment (*n*, %)**				
ACEI/ARB II	99 (60.7)	198 (64.7)	34 (64.2)	***0.0001***
Beta-blockers	105 (64.4)	195 (63.7)	33 (62.3)	*0.940*
ARNI	41 (25.3)	70 (22.9)	11 (20.8)	*0.747*
MRA	68 (41.7)	138 (45.1)	23 (43.4)	*0.773*
SGLT2i	37 (22.7)	77 (25.2)	11 (20.8)	*0.722*
Ivabradine	19 (11.7)	52 (17.0)	8 (15.1)	*0.319*
Digoxin	52 (31.9)	83 (27.1)	18 (34.0)	*0.394*
Loop diuretics	157 (96.3)	282 (92.2)	49 (92.5)	*0.123*
Thiazides	26 (16.0)	46 (15.0)	6 (11.3)	*0.730*
Acetazolamide	3 (1.8)	9 (2.9)	1 (1.9)	*0.738*
Tolvaptan	6 (3.7)	18 (5.9)	2 (3.8)	*0.537*
Potassium supplement	23 (14.1)	49 (16.0)	9 (17.0)	*0.836*
Hypokalemic therapy	6 (3.7)	9 (2.9)	2 (3.8)	*0.885*
Antiplatelet agents	52 (31.9)	107 (35.0)	17 (32.1)	*0.792*
Anticoagulants	97 (59.5)	208 (68.0)	32 (60.4)	*0.172*
OAD (No SGLT2i)	62 (38.0)	135 (44.1)	25 (47.2)	*0.371*
Nitrates	16 (9.8)	46 (15.0)	8 (15.1)	*0.289*
Antiarrhythmic	36 (22.1)	61 (19.9)	10 (18.9)	*0.802*
Statins	97 (59.5)	153 (50.0)	28 (52.8)	*0.125*
Calcium antagonists	41 (25.2)	67 (21.9)	11 (20.8)	*0.655*
Pulmonary vasodilator	3 (1.8)	3 (1.0)	1 (1.9)	*0.691*
Alopurinol	42 (25.8)	67 (21.9)	11 (20.8)	*0.565*

ACEI/ARB-II, angiotensin-converting enzyme inhibitors/angiotensin II receptor blockers; ALT, alanine aminotransferase; ARNI, angiotensin receptor-neprilysin inhibitors; AST, aspartate aminotransferase; CA125, cancer antigen 125; GFR, glomerular filtration rate; HbA1c, glycated hemoglobin A1c; MRA, mineralocorticoid receptor antagonists; NT-proBNP, *n*-terminal pro-brain natriuretic peptide; OAD, oral antidiabetic; TnT, cardiac troponin T; TSAT, transferrin saturation; SGLT2i, sodium-glucose co-trans-porter inhibitors type 2.

**Table 4:** **Analytical and pharmacological HFpEF profile.** Data are expressed as % of cases and median ± interquartile range for non-normally distributed variables (#). *p* < 0.05 was considered statistically significant.

	ID *n* = 110	Treated ID *n* = 234	No ID *n* = 25	*p*
**Laboratory tests on admission analytics (#)**				
Urea (mg/dL)	67.0 (32.0)	63.0 (36.3)	66.0 (42.0)	*0.605*
Creatinine (mg/dL)	0.91 (0.68)	1.04 (0.65)	1.07 (1.28)	*0.262*
GFR (mL/min/1.73 m^2^)	60.0 (57.0)	58.0 (25.0)	57.0 (67.0)	*0.897*
Bilirubin (mg/dL)	1.1 (0.6)	1.1 (0.9)	1.0 (0.5)	*0.833*
AST (U/L)	19.0 (16.1)	17.0 (16.4)	18.5 (18.8)	*0.548*
ALT (U/L)	23.0 (19.0)	18.0 (21.0)	20.5 (23.0)	*0.109*
TnT(u) (ng/mL)	45.3 (48.3)	49.0 (11.3)	49.3 (43.4)	*0.551*
NT-proBNP (pg/mL)	5710 (4905)	5513 (4837)	7953 (7390)	*0.074*
Sodium (mEq/L)	139.0 (6.5)	140.0 (6.0)	139 (4.0)	*0.292*
Potassium (mEq/L)	4.0 (0.6)	4.1 (0.9)	3.9 (0.4)	*0.331*
Hemoglobin (g/dL)	13.0 (4.9)	12.2 (2.0)	12.1 (1.3)	*0.076*
Hematocrit (%)	40.6 (12.7)	38.6 (11.3)	37.3 (11.2)	*0.233*
Uric acid (mg/dL)	8.2 (4.0)	8.1 (4.4)	8.5 (4.6)	*0.900*
Cholesterol-HDL (mg/dL)	41.0 (17.9)	40.0 (15.4)	40.0 (17.7)	*0.865*
Cholesterol-LDL (mg/dL)	77.0 (36.9)	70.0 (34.2)	72.0 (32.6)	*0.224*
Triglycerides (mg/dL)	69.0 (69.0)	65.0 (53.0)	88.0 (74.8)	*0.182*
Ferritin (ng/mL)	103 (64.03)	94.0 (34.1)	406.0 (102.0)	***<0.0001***
TSAT (%)	15.0 (5.2)	12.0 (3.0)	22.0 (2.0)	***<0.0001***
HbA1c (%)	5.8 (1.7)	5.9 (0.7)	5.6 (1.4)	*0.377*
CA125 (U/mL)	50.1 (75.6)	53.0 (73.4)	64.0 (63.2)	*0.694*
**Discharge treatment (*n*, %)**				
ACEI/ARB II	61 (55.5)	131 (60.0)	15 (60.0)	*0.917*
Beta-blockers	66 (60.0)	133 (56.8)	15 (60.0)	*0.839*
ARNI	3 (2.7)	5 (2.1)	1 (4.0)	*0.825*
MRA	38 (34.5)	70 (29.9)	8 (32.0)	*0.688*
SGLT2i	29 (26.4)	54 (23.1)	6 (24.0)	*0.802*
Ivabradine	2 (1.8)	9 (3.8)	1 (4.0)	*0.599*
Digoxin	25 (22.7)	51 (21.8)	6 (24.0)	*0.958*
Loop diuretics	101 (91.8)	218 (93.2)	24 (96.0)	*0.746*
Thiazides	26 (23.6)	51 (21.8)	6 (24.0)	*0.914*
Acetazolamide	1 (0.9)	3 (1.3)	1 (4.0)	*0.477*
Tolvaptan	7 (6.4)	12 (5.1)	2 (8.0)	*0.787*
Potassium supplement	11 (10.0)	12 (5.1)	3 (12.0)	*0.156*
Hypokalemic therapy	10 (6.1)	11 (4.7)	3 (12.0)	*0.157*
Antiplatelet agents	24 (21.8)	48 (20.5)	6 (24.0)	*0.901*
Anticoagulants	67 (60.9)	147 (62.8)	16 (64.0)	*0.929*
OAD (No SGLT2i)	33 (30.0)	68 (29.1)	8 (32.0)	*0.947*
Nitrates	13 (11.8)	21 (9.0)	3 (12.0)	*0.675*
Antiarrhythmic	22 (20.0)	44 (18.8)	3 (12.0)	*0.950*
Statins	59 (5.4)	129 (55.1)	12 (48.0)	*0.786*
Calcium antagonists	41 (37.3)	94 (40.2)	10 (40.0)	*0.874*
Pulmonary vasodilator	2 (1.8)	7 (3.0)	0 (0.0)	*0.576*
Alopurinol	21 (19.1)	54 (23.1)	6 (24.0)	*0.684*

ACEI/ARB-II, angiotensin-converting enzyme inhibitors/angiotensin II receptor blockers; ALT, alanine aminotransferase; ARNI, angiotensin receptor-neprilysin inhibitors; AST, aspartate aminotransferase; CA125, cancer antigen 125; GFR, glomerular filtration rate; HbA1c, glycated hemoglobin A1c; MRA, mineralocorticoid receptor antagonists; NT-proBNP, *n*-terminal pro-brain natriuretic peptide; OAD, oral antidiabetic; TnT(u), cardiac troponin T; TSAT, transferrin saturation; SGLT2i, sodium-glucose co-trans-porter inhibitors type 2.

**Table 5:** **Effect of FCM treatment on morbidity and mortality.** Data are expressed as % of cases. Assignment to each group was exclusive (Patients who died were counted in the deceased group. Surviving hospitalized patients were counted in the re-admission group. Patients seen in the emergency unit were included in neither the hospitalized nor deceased group). In the case of more than one visit, only one was counted, since the calculation was based on the percentage of patients who presented the event). *p* < 0.05 was considered statistically significant.

**Heart Failure with *Reduced* Ejection Fraction**				
	**ID** ***n* = 162**	**Treated ID** ***n* = 306**	**No ID** ***n* = 53**	***p***
**Emergency visits (*n*, %)**	29 (17.9)	39 (12.7)	3 (5.7)	0.062 ^a^ 0.132 ^b^
**Re-admission for HF (*n*, %)**	43 (26.5)	75 (24.5)	11 (20.8)	0.690 ^a^ 0.630 ^b^
**All-cause mortality (*n*, %)**	16 (9.9)	23 (7.5)	4 (7.5)	0.664 ^a^ 0.379 ^b^
**Combination (*n*, %)**	88 (54.3)	137 (44.8)	18 (34.0)	**0.021** ^a^ **0.049** ^b^
**Heart Failure with *Preserved* Ejection Fraction**				
	**ID** ***n* = 110**	**Treated ID** ***n* = 234**	**No ID** ***n* = 25**	***p***
**Emergency visits (*n*, %)**	27 (24.5)	50 (21.4)	5 (20.0)	0.773 ^a^ 0.510 ^b^
**Re-admission for HF (*n*, %)**	30 (27.3)	51 (21.8)	5 (20.0)	0.492 ^a^ 0.264 ^b^
**All-cause mortality (*n*, %)**	11 (10.0)	22 (9.4)	2 (8.0)	0.951 ^a^ 0.861 ^b^
**Combination (*n*, %)**	68 (61.8)	123 (52.6)	12 (48.0)	0.210 ^a^ 0.107 ^b^

^a^ Comparison between the three groups; ^b^ ID vs. treated ID.

**Table 6:** FCM treatment effect on risk reduction and number of patients to treat in subjects with iron deficiency.

**Heart Failure with *Reduced* Ejection Fraction**					
	**ID**	**Treated ID**	**ARR**	**RRR**	**NNT**
Emergency visits (*n*, %)	29 (17.9)	39 (12.7)	5%	29%	19.4
Re-admission for HF (*n*, %)	43 (26.5)	75 (24.5)	2%	8%	49.2
All-cause mortality (*n*, %)	16 (9.9)	23 (7.5)	2%	24%	42.4
Combination (*n*, %)	88 (54.3)	137 (44.8)	10%	18%	10.5
**Heart Failure with *Preserved* Ejection Fraction**					
	**ID**	**Treated ID**	**ARR**	**RRR**	**NNT**
Emergency visits (*n*, %)	27 (24.5)	50 (21.4)	3%	13%	31.5
Re-admission for HF (*n*, %)	30 (27.3)	51 (21.8)	5%	20%	18.3
All-cause mortality (*n*, %)	11 (10.0)	22 (9.4)	1%	6%	167.1
Combination (*n*, %)	68 (61.8)	123 (52.6)	9%	15%	10.8

ARR: absolute risk reduction. NNT: number needed to treat; RRR: relative risk reduction.

## Data Availability

Not applicable.
